# Bilan d’activité de l’unité de greffe de moelle osseuse du Centre Hospitalier Universitaire Mohamed VI de Marrakech, Maroc: 2012-2018

**DOI:** 10.11604/pamj.2021.39.176.29029

**Published:** 2021-07-06

**Authors:** Mehdi Loukhnati, Fatima Ezzahra Lahlimi, Illias Tazi

**Affiliations:** 1 Service d´Hématologie Clinique et Greffe de Moelle, Centre Hospitalier Universitaire Mohammed VI, Université Cadi Ayyad, Marrakech, Maroc

**Keywords:** Cellules souches, autogreffe, allogreffe, conditionnement, Marrakech, Stem cells, autograft, allograft, conditioning, Marrakech

## Abstract

**Introduction:**

la greffe de cellules souches hématopoïétiques (GCSH) fait partie de l´arsenal de l´immunothérapie cellulaire à but curatif, elle est utilisée dans le traitement de plusieurs affections hématologiques malignes et non malignes ainsi que d´autres pathologies extra-hématologiques. Décrite depuis 1950 et introduite au Maroc depuis les années 2000, la GSCH reste encore jeune dans notre contexte avec plusieurs obstacles juridiques, financiers et organisationnels. L´objectif de notre travail est de rapporter l´expérience en matière de la greffe des cellules souches hématopoïétiques au sein du service d´hématologie clinique et de greffe de moelle osseuse du centre hospitalo-universitaire (CHU) Mohammed VI de Marrakech, étant un exemple d´un service des pays en voie de développement.

**Méthodes:**

nous avons mené une étude rétrospective descriptive sur une durée de 7 ans.

**Résultats:**

soixante-six GCSH ont été réalisées, le myélome multiple représente la première indication dans notre série avec 30 autogreffes. Dans notre série, Le taux de mortalité avoisine les 20%, celui de rechute est à 23% et celui des complications est à 38%. Nos résultats malgré les difficultés restent encourageants, avec un recul assez important.

**Conclusion:**

des efforts restent à fournir pour améliorer les résultats de cette thérapie.

## Abstract (en)

**Introduction:**

hematopoietic stem cell transplantation (HSCT) is part of the cellular immunotherapy arsenal. It is used in the treatment of several malignant and non-malignant hematological conditions as well as other extra-hematological diseases. HSCT has been described since 1950 and introduced in Morocco since the 2000s. GSCH is still little used in our context due to several legal, financial and organizational barriers. The purpose of this study is to report the experience of the Bone Marrow Transplant Department of the Marrakech’s Mohammed VI University Hospital with hematopoietic stem cell transplantation, is one of the Hospital Departments in developing countries.

**Methods:**

we carried out a descriptive retrospective study over a period of 6 years.

**Results:**

during the study period, sixty-six HSCT were performed. Multiple myeloma was the main indication for HSCT in our case series (30 patients with autografts). In our case series, mortality rate was around 20%, relapse rate was 23% while complications rate was 38%. Despite the challenges, our results were encouraging during the long follow-up period.

**Conclusion:**

efforts still need to be made to improve therapy results.

## Introduction

La greffe de moelle aussi bien autologue qu´allogénique permet de guérir un nombre considérable de maladies hématologiques, génétiques et immunologiques. Cette thérapeutique est assez courante dans le monde et le nombre de centre de greffe est en nette augmentation dans les pays développés. En revanche dans les pays en voie de développement, et en raison du manque de moyens matériels, humains et financiers, cette modalité thérapeutique demeure l´apanage de quelques structures hospitalières et le nombre réalisé reste limité en dépit de la volonté politique et de la motivation des équipes soignantes.

Malgré sa complexité, cette procédure a vu le jour au Maroc en 2004 au niveau du centre d´hématologie de Casablanca [1], et fait désormais partie du paysage médical quotidien dans divers centres publiques que privés du royaume. Il nous a paru intéressant d´étudier les données du service d´hématologie de Marrakech sur une période de sept ans afin de dégager les aspects positifs et aussi négatifs pour une prise en charge optimale des malades, tout en discutant les modalités d´amélioration et l´instauration d´autres techniques de transplantation médullaire dans cette unité.

## Méthodes

**Type d´étude:** nous avons mené une étude rétrospective descriptive sur une durée de sept ans allant au mois de janvier 2012 au mois de décembre 2018 au sein du service d´hématologie clinique et de greffe de moelle au Centre Hospitalier Universitaire à Marrakech au Maroc.

### Population cible

**Critères d´inclusion:** nous avons inclus dans notre étude tous les patients suivis et hospitalisés au service d´hématologie, centre onco-hématologie, CHU Mohammed VI de Marrakech, ou suivis dans d´autres structures publiques ou privées ne disposant pas de plateforme de greffe et dont l´indication de greffe a été validée en réunion de concertation pluridisciplinaire. L´indication de l´autogreffe a été posée chez les sujets atteints de myélome multiple en rémission complète et chez les sujets présentant un lymphome Hodgkinien ou non Hodgkinien réfractaire ou en rechute précoce. L´indication de l´allogreffe a été principalement chez les sujets ayant une aplasie médullaire très sévère, une leucémie myéloïde chronique résistante aux inhibiteurs de tyrosine kinase, un déficit immunitaire ou une béta-thalassémie majeure.

**Critères d´exclusion:** nous avons exclu de notre étude les patients non éligibles à la greffe principalement les sujets âgés de plus de 65 ans, ou ayant une lourde comorbidité, et en cas d´absence de rémission pour les hémopathies malignes.

**Recueil des données:** cette étude a été basée sur l´exploitation des dossiers cliniques, en recueillant les principales données sur une fiche d´exploitation

**Les données épidémiologiques:** âge, sexe, consanguinité, couverture sociale.

**Les données cliniques:** l´évaluation clinique pratiquée à l´entrée de l´étude, puis au début de chaque cycle thérapeutique, a comporté l´appréciation des: symptômes, performance statuts et examen clinique complet.

**Les données paracliniques:** le bilan paraclinique diffère selon chaque pathologie, il comporte les éléments nécessaires au diagnostic, pronostic ainsi qu´au suivi.

### Les données thérapeutiques

**Activité d´autogreffe:** la mobilisation des cellules souches hématopoïétiques était faite par les facteurs stimulants les colonies de granulocytes (G-CSF): le lénograstim a été administré seul à la dose de 10 µg/kg/j en cas de MM et 15 µg/kg/j en cas de lymphome. La mobilisation par une chimiothérapie à base de cyclophosphamide haute dose était aussi adopté chez certains patients avec lymphome. La collecte des cellules souches était faite le 5^e^ jour de mobilisation par appareil Spectra Optia, une collecte réussie est représentée par taux de CD34 supérieur ou égal à 2 x 10^6^ par kilogramme du receveur après une à deux voire trois collectes.

Les greffons étaient conservés soit à +4°C en cas de myélome multiple ou en azote liquide à -80°C en cas de lymphome En cas MM, le conditionnement pré-greffe s´est fait par le Melphalan haute dose (200mg/m^2^), 24 heures avant la réinjection des CSP. Pour les patients atteints d´un lymphome, le conditionnement pré-greffe se faisait selon le protocole BEAM (Bicnu, etoposide, aracytine, melphalan) ou BendaEAM (Bendamustine, etoposide, aracytine, melphalan). Pour prévenir la mucite, tous nos patients ont reçu le Caphosol en bain de bouche 6 fois par jour et des glaçons à sucer durant le conditionnement. Tous les patients ont reçu une prophylaxie anti-infectieuse à base de ciprofloxacine 500mg x 2 par jour et recevait le lénograstim 5µg/kg/j à partir du cinquième jour de la greffe.

**Activité d´allogreffe:** le prélèvement des cellules souches, médullaire ou périphérique était prélevé le même jour de la greffe, les donneurs sains d´allogreffe ont été mobilisés par lénograstim à 5µg/kg/j pendant 4 à 5 jours. Pour les patients atteints d´une aplasie médullaire, le conditionnement était à base de cyclophosphamide associé au sérum anti lymphocytaire du lapin. Les patients atteints d´un déficit immunitaire combiné sévère n´ont pas reçu de conditionnement pré-greffe. Pour les autres pathologies une combinaison d´une polychimiothérapie (Busulfan, fludarabine, thiotepa) était la base du conditionnement.

La prophylaxie de la réaction du greffon contre l´hôte GVH a consisté en l´administration de la ciclosporine associée selon les cas au methotrexate à faible dose. La prophylaxie anti-infectieuse était à base de ceftriaxone, fluconazole et acyclovir, et le cotrimoxazole à partir du J21. La prise en charge des manifestations de la GVH reposait sur le traitement symptomatique avec une augmentation est dose de la ciclosporine associée ou non à des bolus de méthylprednisolone.

**Transfusion:** la transfusion sanguine se faisait par des culots globulaires déleucocytés, compatibilisés, phénotypés et irradiés, La transfusion plaquettaire se faisait aussi par des unités plaquettaires d´aphérèse (CPA) irradiées.

**Traitement anti-infectieux:** la stratégie thérapeutique en cas de neutropénie fébrile dépendait de l´écologie du service, en se basant aussi sur les recommandations internationales.

**Les données évolutives:** nous avons utilisé principalement les critères d´évolutivités correspondantes à chaque pathologie.

**Analyse statistique:** l´analyse statistique des données a été réalisée à l´aide du Microsoft Office Excel® 2013. Les variables qualitatives ont été exprimées en pourcentage et les variables quantitatives ont été exprimées par les moyennes et les limites. Afin de comparer nos résultats avec ceux de la littérature, nous avons procédé à une recherche bibliographique, l´analyse des ouvrages et articles traitant la transplantation des cellules souches.

**Ethique:** pour respecter le secret médical, nous avons gardé l´anonymat des malades.

## Résultats

**Etat des lieux:** l´unité de greffe du Centre Hospitalier Universitaire Mohammed VI de Marrakech est constituée de six chambres individuelles à pression négative et flux laminaire. Le staff médical est constitué de deux professeurs, un spécialiste en hématologie et un médecin résident en cours de formation, l´équipe infirmière est composé d´un infirmier major et sept infirmiers polyvalents.

Le service d´hématologie abrite une unité dédiée à l´activité d´aphérèse sous la responsabilité du service de la transfusion sanguine, l´équipe est composée de 2 médecins et 2 infirmiers, elle assure le besoin des prélèvements des cellules souches et les plaquettes d´aphérèse. Depuis 2018, le prélèvement de cellules souches est assuré par le service de thérapie cellulaire qui assure en plus le traitement et la conservation des greffons. L´irradiation des produits sanguins labiles s´effectuaient au service de radiothérapie. Depuis 2019, le service dispose d´un irradiateur de plaquettes.

**Le cout de greffe à Marrakech:** le système d´assurance au Maroc est réparti en régime médical de base assuré par l´état (Régime d´assistance médical dit RAMED), d´assurance publique ou privée. L´ensemble du système, couvre l´activité de greffe avec un taux de couverture qui varie entre 90% à 100%. Le cout d´une autogreffe varie entre 12 000 et 19 000 euro, pour l´allogreffe le cout avoisine 90 000 euro, la prise en charge est régie par un système forfaitaire et inclut tous les éléments de la prise en charge: hospitalisation, bilans, collecte et conservation du greffon, médicaments, support transfusionnel et suivi post greffe.

### Activité d´autogreffe des cellules souches hématopoïétiques

#### Caractéristiques des patients

**Myélome multiple (MM)**: c’est la première indication d´autogreffe de cellules souches hématopoïétiques. Trente patients (45%) ont été autogreffés pour un MM, le Tableau 1 résume les caractéristiques de ces patients. Vingt et un (21) patients (n=70%) ont reçu une seule ligne de chimiothérapie: le protocole CDT (cyclophosphamide, dexamethasone et thalidomide) a été utilisé chez 17 malades, le protocole VAD (vincristine, adriamycine et dexamethasone) a été utilisé chez un malade et le protocole VTD (Bortezomib, thalidomide et dexamethasone) chez un autre. L´évaluation de la réponse a respecté les recommandations de l´international myeloma working group [2]. Deux lignes de chimiothérapie étaient utilisées chez 7 malades (23%), avec des régimes contenant du Bortézomib ou lénalidomide et 3 lignes de chimiothérapie ont été utilisés chez 2 malades (7%). La cryoconservation a été utilisé chez 2 malades (7%) alors que chez 28 malades (93%) le greffon a été conservé à +4°C.

**Tableau 1:** caractéristiques des patients atteints de myélome

Caractéristiques (n=30)	valeur
Médiane d'âge en année	49 (43-63)
Sex ration	22/8
Couverture sociale	
Public	20 (66.6%)
Privée	10 (33.3%)
**ISS**	
I	10 (33.3%)
II	10 (33.3%)
III	10 (33.3%)
Insuffisance rénale	3 (10%)
**Protocole initial**	
1 une ligne de chimiothérapie	21 (70%)
2 lignes de chimiothérapie	7 (23%)
3 lignes de chimiothérapie	2 (7%)
**Rémission (selon les critères IMWG [2])**	
RC	10 (33.3%)
VGPR	10 (33.3%)
RP	10 (33.3%)
Délai d'attente de la greffe en mois	7.5 (1-16)
**Jour de collecte**	
1 jour	28 (93%)
2 jours	2 (7%)
Richesse du prélèvement CD34 en 106/kg	7.42 (2-16)
**Conditionnement**	
Melphalan 200 mg/m^2^	29 (96.6%)
Melphalan 140 mg/m^2^	1 (3.4%)

ISS: international scoring système, RC: rémission complète, VGPR: très bonne réponse partielle, RP: réponse partielle, IMWG: international myeloma working group [2]

**Lymphome:** quatorze patients ont été colligés, le Tableau 2 résume les caractéristiques des patients. Chez 2 patients atteints de lymphome (14,2%), la mobilisation a été faite par une chimiothérapie de mobilisation à base de cyclophosphamide haute dose associé au G-CSF. La durée de cryoconservation variait entre 7 jours et 12 mois. La richesse du greffon après la décongélation n´a pas été évaluée.

**Tableau 2:** caractéristiques des patients atteints de lymphome

Caractéristiques (n=14)	valeur
Age médian en année	31 (16-56)
Sex ration	4/10
**Type de lymphome**	
LH	9 (64%)
DLBCL	2 (14%)
Richter	1 (7%)
MCL	1 (7%)
T/NK	1 (7%)
Ligne de chimiothérapie1ligne	1 (7%)
2 lignes	12 (86%)
4 lignes	1 (7%)
**Type de rémission**	
RC radiologique	9 (64%)
RP radiologique	5 (36%)
Délai d'attente de la greffe en mois	7 (2-13)
**Jour de collecte**	
1 jour	12 (85.7%)
2 jours	2 (4.3%)
CD34 en 106/kg	5.2 (2-17)
**Conditionnement**	
BEAM	13 (93%)
BendaEAM	1 (7%)

LH: lymphome de Hodgkin; DLBCL: lymphome B à grande cellules; MCL: lymphome du manteau RC: rémission complète; RP: rémission partielle; BEAM: Bicnu, etoposide, aracytine et melphalan BendaEAM: Bendamustine, etoposide, aracytine et melphalan

**Evolution:** les complications aiguës étaient dominées par les effets secondaires gastro-intestinaux: 88,6 % des patients ont présenté des nausées et/ou des vomissements. La neutropénie fébrile a survenu chez 79.5% des patients avec un foyer pulmonaire clinique chez 18,18% et digestive chez 27,27%. Environ 6,81% des patients ont eu recours aux antifongiques pour aspergillose pulmonaire ou candidémie, alors que l´utilisation d´acyclovir a été nécessaire chez un malade réactivant un herpès labial. Le Tableau 3 résume l´évolution des patients.

**Tableau 3:** évolution des patients autogreffés

Caractéristiques	Valeur
Mucite	
Mucite grade 1-2	36 (81.8%)
Mucite grade 3-4	8 (18.2%)
Nausées –vomissements (tous grade)	39 (88.6%)
Diarrhées	14 (31.8)
Neutropénie fébrile	34 (77.3%)
Alimentation parentérale	8 (18.2%)
Moyenne des jours	4 (2-7)
Echec de greffe	0
Récupérations des polynucléaires en jours	8 (5-19)
Récupérations des plaquettes en jours	4 (0-27)
Transfusion en culot globulaire	2 (0-6)
Transfusion en plaquettes (pool)	2 (0-8)
Transplant related mortality	1 (2.3%)
Durée d'hospitalisation en jours	20 (13-39)

La neutropénie fébrile était présente chez 34 malades (77,3%), un foyer clinique était présent chez 6 malades (13,6%) représenté par une pneumonie chez 3 malades (6,8%) et une diarrhée chez 3 autres (6,8%). L´hémoculture était positive chez 12 malades (27,3%), les coccis gram positif représentaient 68% et les grams négatifs 32%. Le staphylocoque à coagulase négative a représenté 37%, suivi de staphylocoque aureus et streptocoque 6% chacun. Un décès aigu (2,3%) est survenu chez un patient suivi pour un lymphome suite à une hémorragie digestive cataclysmique. La durée moyenne d´hospitalisation était de 20 jours (13 à 39 jours).

Le suivi à moyen et à long terme était réalisé chez 75% des malades, 25% des patients ont été perdu de vue après leur première consultation post greffe. Le Tableau 4 résume les caractéristiques de suivi. Cinq (5) patients (11,3%) ont rechuté après l´autogreffe, dont deux (4,54) en rémission partielle après une 2^e^ ligne de chimiothérapie et en attente d´une 2^e^ autogreffe.

**Tableau 4:** suivi des malades

Caractéristiques	Valeur
Suivi régulier	33 patients (75%)
Perdu de vue	11 patients (25%)
Durée de suivi en mois	22 (9-50)
Rémission complète	28 patients (63,7%)
Rechute	5 patients (11,3%)
Délai en mois	8 (5-14)
Décès	3 patients (6,81%)
Rémission après chimiothérapie	2 patients (4,54%)

**Activité d´allogreffe des cellules souches hématopoïétiques:** 22 malades ont bénéficié d´une allogreffe CSH avec une répartition égale du sexe (sexe ratio 1/1) dont 63% était âgé de moins de 15 ans. L´âge moyen était de 17 ans (2 à 46 ans). Les caractéristiques des patients allogreffés sont résumées dans le Tableau 5. Les complications à court et moyen terme sont comme suit: hémolyse aiguë à J0 chez 4.35% due à une incompatibilité ABO; maladie veino-occulsive chez 8.7% des patients résolutive sous traitement symptomatique; choc septique chez 17,4% des patients; pancreatite aiguë chez 4,35% des cas, probablement d´origine médicamenteuse (Norgestrel); microangiopathie thrombotique (MAT) chez 4.35% des cas; thrombose de la veine illiaque interne chez 4,35% des cas; rejet primaire de greffon chez 4,35% des cas; GVH cutanée chez 8,7% des patients; décès précoce est survenu chez 30,4% des patients (choc septique, MAT, choc hémorragique).

**Tableau 5:** caractéristiques des patients allogreffes

Caractéristiques (n=22)	valeur
**Pathologies sous-jacentes:**	
Aplasie médullaire idiopathique	7 (31.9%)
Leucémie aigüe myéloïde	2 (9%)
Leucémie aigue lymphoblastique	2 (9%)
Leucémie myéloïde chronique	3 (13.7%)
Béta thalassémie	4 (18.2%)
Déficit immunitaire combiné sévère	2 (9%)
Déficit en HLA classe II	2 (9%)
Age médian en année	17 (2-46)
Sexe ratio	1/1
**Donneurs**	
Géno-identique	22 (100%)
Phéno-identique	0
Haplo-identique	0
**HLA**	
10/10	22 (100%)
8-9/10	0
**Greffon**	
Médullaire	19 (82.6%)
CSP	4 (17.4%)
Taux de cellules mononuclées en 108 par kilogramme du receveur	3.2 (2.1-6)
CD34 en 106 par kilogramme du receveur	5.2 (4.3-11)

La GVH chronique est apparue chez 2 malades, la première à type de GVH pulmonaire et la deuxième à type GVH cutané grade IV, causant 2 décès tardifs. Le suivi est réalisé chez tous les patients en consultation spécialisée. Environ 60,8% des patients (n=14) sont toujours sous surveillance régulière avec un recul moyen de 48 mois (15 à 72 mois). La rémission complète est survenue chez 52,2% des patients, avec une rechute chez 8,7% des patients.

## Discussion

Le Maroc est un pays africain en voie de développement avec un revenu moyen estimé à 250 euro par habitant et par mois [3]. Il comporte plusieurs systèmes de couverture médicale: le RAMED (Régime d´Assitance Médicale) destiné aux populations les plus démunies, les assurances publiques destinées aux fonctionnaires de l´état et les assurances privées. L´activité de greffe qu´elle soit autologue ou allogénique est prise en charge par les différentes structures d´assurance avec un plafond de couverture de 90 à 100%.

Le Maroc dispose de 7 centres hospitalo-universitaires publics et 3 centres privés. L´activité de greffe de moelle au secteur publique est réalisée uniquement par 3 centres à Marrakech, Casablanca et à Rabat au niveau de l´hôpital militaire d´instruction, elle comporte aussi bien l´activité autologue qu´allogénique. Les centres hospitalo-universitaires privés, ainsi que quelques cliniques libérales du royaume sont dotés de plateaux techniques permettant pour le moment de faire qu´une activité de greffe de moelle autologue.

La première autogreffe de moelle a été réalisée en 2004 à Casablanca pour un malade présentant un myélome multiple, puis à Marrakech en 2012 pour un malade présentant la même pathologie en utilisant cette fois la technique sans congélation [1, 4]. L´activité d´allogreffe a vu le jour en 2016 pour un enfant présentant un déficit immunitaire combiné sévère, elle a été réalisée sans conditionnement [5]. Dans la ville de Marrakech, 66 sujets ont été greffés entre la période de 2012 à 2018 comportant aussi bien des autogreffés et des allogreffés.

À Rabat au niveau de l´hôpital militaire, 43 autogreffes chez des patients porteurs de MM ont été réalisées entre 2015 et 2017 [5]. En Algérie, entre 2009 et 2016, 352 patients ont bénéficié d´une autogreffe, dont 240 myélomes, 82 cas de lymphomes hodgkiniens et 30 cas de lymphomes non hodgkiniens [6]. Au Pakistan, ente 1995 et 2005, plus de 340 TCSH ont été réalisées [7]. Dans notre série, à Marrakech, l´autogreffe des CSH et l´allogreffe des CSH représentent respectivement 66,3% et 33,7% de l´activité de la GCSH, ce qui est semblable aux pourcentages rapportés dans les séries algériennes [6]. Il faut de ce fait souligner que malgré les efforts fournis pour le développement de cette thérapeutique, la GCSH ne représente qu´une minorité des thérapeutiques utilisées en hématologie et ceci est lié en particulier aux problèmes financiers répétitifs, au retard des démarches administratives, au délai d´attente des malades en particulier les plus urgents d´entre eux, la rupture répétée et prolongée de certains médicaments de conditionnements en raison de la difficulté d´approvisionnement par le ministère de la santé et aussi la difficulté d´assurer une continuité de cette activité en raison de la demande accrue des indications thérapeutiques posant le problème de rechute ou de décès avant même la réalisation de greffe. Les patients autogreffés pour MM avaient un âge inférieur à 65 ans, cette donnée rejoint ce qui est rapporté dans la littérature [8, 9].

La mobilisation des cellules souches par G-CSF est le gold standard à la dose de 10 µg/kg/jour en sous cutané pendant 4 à 5 jours. L´utilisation de la chimiothérapie en mobilisation reste une autre option valide dans la littérature mais rarement utilisé en cas de MM [10, 11]. Aucun de nos patients porteurs du MM n´a reçu une chimiothérapie pour la mobilisation. Le taux du CD34 moyen était à 7.42 x 10&sup6;/Kg, ce qui reste supérieure à la moyenne minimale requise [10, 11]. La chimiothérapie de conditionnement reposait sur le Melphalan à haute dose comme ce qui est utilisé par les autres équipes [8-11]. Dans notre série, nous avons opté pour l´autogreffe de cellules souches sans congélation chez les malades myélomateux. Cette technique est de plus en plus utilisée dans les pays en voie de développement en raison du coût moins élevé et les résultats satisfaisants rapportés par plusieurs équipes avec une durée moyenne de la sortie d´aplasie de 7 à 10 jours [3, 5, 6, 8, 9].

L´autogreffe chez les patients atteints de lymphome est indiquée en cas de rechute, en cas d´une maladie réfractaire et après d´obtention d´une rémission par une chimiothérapie de rattrapage. Elle est rarement proposée comme un traitement de consolidation en première ligne, comme en cas du lymphome du manteau [12-17]. La mobilisation était à base du G-CSF associée ou non à une chimiothérapie. Cette association était utilisée chez 14% de nos malades qui ont reçu plus de 2 lignes de chimiothérapie. L´utilisation du cyclophosphamide à la dose de 3g/m2 est utilisée pour la mobilisation par certaines équipes [17]. Le régime de conditionnement repose sur le protocole BEAM utilisé chez 93% de nos malades. L´utilisation de la bendamustine a été indiquée chez un malade en raison de non disponibilité de la carmustine à l´échelle nationale au cours d´une période donnée. Le Tableau 6 résume les principales caractéristiques de notre série en les comparants à certaines données de la littérature [3, 6, 18].

**Tableau 6:** séries de la littérature

Auteur	N	CD34 106/kg	Récupération des polynucléaires en jours	Récupérations des plaquettes en jours	TRM-100 jours
Bakadja *et al*. (2017) [6]	240	5.7 (2.3-13.2)	10 (6-17)	13 (9-13)	1.3%
Bakadja *et al*. (2017) [6]	112	3.66 (3.3-9.7)	13 (9-14)	14 (10-37)	2%
Bellaoui *et al*. (2010) [3]	87	7.1 (2-50)	13 (6-49)	21 (7-94)	-
Jennane *et al*. (2020) [18]	55	4.5 (2-12.2)	12 (7-19)	14 (9-32)	3.6%
Notre série	44	7.42 (2-17)	8 (5-19)	4 (0-27)	2.3%

TRM (transplant related mortality)

L´activité de l´allogreffe est lourde dans notre contexte, vu le cout élevé, l´absence de moyens pour la prise en charge initiale et le suivi à moyen et à long terme. Comme d´autres pays en voie de développement, l´absence et les ruptures des moyens pour une prise en charge optimale représentent l´obstacle majeur pour sa réalisation. Nous avons été aidés pour nos premières allogreffes par l´équipe de *Cure2children*, qui était présente sur place dans notre formation. L´indication de la première allogreffe réalisée dans notre unité portait sur un enfant âgé de 10 ans qui présentait une LAL B avec rechute neurologique, l´évolution était fatale par une réaction du greffon contre l´hôte résistante à toute thérapeutique. La prise en charge de nos malades se faisait selon des protocoles issus de guidelines internationaux [19].

L´indication majeure dans notre série était l´aplasie médullaire idiopathique dans le tiers des cas, suivie par les leucémies aiguës et chroniques puis par les déficits immunitaires. Nous avons également allogreffé deux enfants thalassémiques. Ces données varient selon les données de plusieurs centres [6, 7, 20]. La prophylaxie de GVH reposait sur la cyclosporine associée ou non au méthotrexate. En raison du faible nombre de malades allogreffés répertoriés dans notre série et la disparité des pathologies étudiées, nous n´avons pas pu comparer nos résultats à ceux de la littérature. Le suivi régulier des malades est fait par les médecins traitants dédiés à la greffe. Il est tout de même important de souligner l´importance de la formation continue de nos médecins et infirmiers dans des centres de greffe conventionnés avec notre structure pour acquérir plus d´expérience. En effet, certains membres de notre équipe ont effectué des stages courts ou prolongés dans des services hospitaliers en France ou en Italie, ou dans des pays en voie de développement ayant des conditions similaires aux nôtres.

Le nombre de malades inclus dans notre étude reste très limité et peut être source d´imprécisions, donc l´analyse statistique ne peut pas se faire dans les règles de l´art, ce qui constitue un biais statistique Concernant les perspectives d´avenir, une banque du sang du cordon a été mise en place en 2018 dans notre centre hospitalier pour procéder prochainement à l´allogreffe à partir du sang placentaire. Nous nous acharnons également pour mettre en place la transplantation haplo-identique qui verra le jour au cours de l´année 2021, ainsi que nous envisagerons d´élargir les indications des autogreffes aux pathologies auto-immunes.

## Conclusion

L´activité de greffe des CSH à Marrakech est en pleine évolution, avec des résultats de plus en plus encourageants. Actuellement, nous disposons d´un circuit de prise en charge bien organisé avec une équipe motivée. Le plus souvent, l´approvisionnement en médicament reste un obstacle majeur pour cette thérapeutique.

### Etat des connaissances sur le sujet

- Traitement d´appoint en hématologie clinique;
- Développement des connaissances ces dernières années;
- Diminution des effets secondaires.

### Contribution de notre étude à la connaissance

- Exemple de greffe dans un pays en voie de développement;
- Difficultés rencontrées au cours des procédures;
- Comparaison des résultats avec des centres similaires.
